# Handedness modulates proprioceptive drift in the rubber hand illusion

**DOI:** 10.1007/s00221-018-5391-3

**Published:** 2018-11-08

**Authors:** Harriet Dempsey-Jones, Ada Kritikos

**Affiliations:** 1 University College London, London, UK; 2 University of Oxford, Oxford, UK; 3 University of Queensland, Brisbane, Australia

**Keywords:** Rubber hand illusion, Somatosensory, Visual, Multisensory integration, Hand dominance, Proprioceptive drift

## Abstract

Preference for use of either the left or right hand (‘handedness’) has been linked with modulations of perception and sensory processing—both of space and the body. Here we ask whether multisensory integration of bodily information also varies as a function of handedness. We created a spatial disparity between visual and somatosensory hand position information using the rubber hand illusion, and use the magnitude of illusory shifts in hand position (proprioceptive ‘drift’) as a tool to probe the weighted integration of multisensory information. First, we found drift was significantly reduced when the illusion was performed on the dominant vs. non-dominant hand. We suggest increased manual dexterity of the dominant hand causes greater representational stability and thus an increased resistance to bias by the illusion induction. Second, drift was generally greatest when the hand was in its habitual action space (i.e., near the shoulder of origin), compared to when it laterally displaced towards, or across the midline. This linear effect, however, was only significant for the dominant hand—in both left- and right-handed groups. Thus, our results reveal patterns of habitual hand action modulate drift both within a hand (drift varies with proximity to action space), and between hands (differences in drift between the dominant and non-dominant hands). In contrast, we were unable to find conclusive evidence to support, or contradict, an overall difference between left- and right-handers in susceptibility to RHI drift (i.e., total drift, collapsed across hand positions). In sum, our results provide evidence that patterns of daily activity—and the subsequent patterns of sensory input—shape multisensory integration across space.

**Electronic supplementary material:**

The online version of this article (10.1007/s00221-018-5391-3) contains supplementary material, which is available to authorized users.

## Introduction

Humans, unlike most other mammals, show a strong preference for use of one hand over the other (Annett 2004; Bryden et al. 2000), especially when executing unimanual tasks. This preference is usually for the right hand, with around 90% of the population being right dominant (Oldfield 1971). Direction of handedness (left vs. right preference) is associated with differences in brain structure and function: e.g., greater lateralisation of brain function in right- compared with left-handers (Steinmetz et al. 1991), linked with smaller corpus callosum size (Witelson 1985).

These brain differences are reflected in variations in perception and cognitive processing between handedness groups (reviewed in Hach and Schütz-Bosbach 2010). In visual spatial perception tasks, for example, right-handers show an exaggeration of the normal leftward spatial bias demonstrated in line bisection tasks, compared with left-handers (Sampaio and Chokron 1992). In processing body-related information, right-handers show an overestimation of the right side of their body compared with left—an asymmetry not seen in left-handers (Hach and Shütz-Bosbach 2010; also see Hoover et al. 2016; Christman et al. 2007 for other handedness-related perceptual differences; Nicholls et al. 2010; Johnston et al. 2010 for cognition related differences).

Here we wished to investigate whether multisensory integration also varies as a function of handedness. Specifically, we ask whether handedness affects the way visual and somatosensory information about the body is combined to contribute to a coherent sense of body position. To do so, we employed a multisensory illusion known to provide a useful index of such integration processes, the rubber hand illusion induction (RHI; Botvinick and Cohen 1998). In this paradigm, the participant’s own hand is hidden and a rubber hand is placed in front of them, in an anatomically plausible position. This creates an illusory spatial disparity between the seen and felt position of the hand. Visual and proprioceptive position information is integrated, causing a shift in felt position of the real hand towards the visual position of the rubber hand (Botvinick and Cohen 1998; Costantini and Haggard 2007; Dempsey-Jones and Kritikos 2014, 2017; Rohde et al. 2011; Tsakiris and Haggard 2005), as well as causing subjective changes (not assessed here, see “Methods”). This change in position estimation, termed proprioceptive ‘drift’, reflects integration of the sensory inputs that is weighted by the reliability of the information being integrated (optimal integration theory: Ernst and Banks 2002; Ernst and Bulthoff 2004).

### The current study

Given the established link between integration and the reliability of sensory inputs, we predicted that following RHI induction we would see less drift for the dominant hand compared with the non-dominant hand. This was anticipated because, consistent with the optimal integration theory, greater stability of the sensorimotor representation of the dominant hand (Barnsley and Rabinovitch 1970; Ni Choisdealbha et al. 2011) should lead to a higher weighting for proprioception during integration. This would cause a greater resistance to the felt position being shifted by the false visual information during illusion induction (see “Discussion” for further interpretation and alternate mechanistic explanations).

Second, various studies have investigated how left- and right-handers may vary in perception and multisensory integration due to documented differences in brain laterality or interhemispheric connectivity (see above). In contrast, here we wished to investigate how dominance might shape integration and perception because of the way it constrains our manual interactions with the world. That is, how handedness causes our hand actions to be more or less lateralised towards either the left or right side of space. Thus, in the current study we look at how drift varies across and between the left and right hemispace, as a function of hand dominance.

**Fig. 1:**
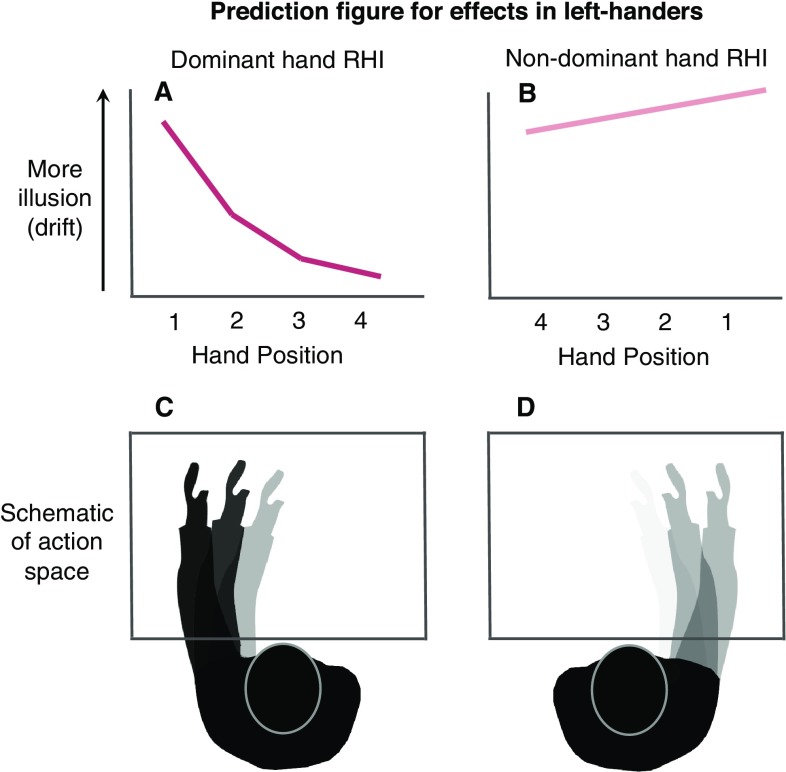
**a, b** Predictions for the relationship between handedness and drift—example for left-handers (note: the inverse pattern between hands is predicted for right-handers). For both hands, we predicted maximal drift in the habitual action space of the arm, i.e., when the hand was positioned near its shoulder of origin (more details in text). **a** For the dominant (left) hand, we anticipated a steep drop in drift from position one to four. For the non-dominant (right) hand, we predicted limited spatial modulation of drift. The bottom panels represent a schematic of the action space of the two hands as a function of dominance. **c** The dominant hand is shown in darker tones as its more frequent use in daily action, was predicted to cause the steeper action-based modulation of drift across space (shown in **a**). **d** This is in contrast to the non-dominant hand that has a less defined action space and thus results in limited drift modulation. *Pos. 1* shoulder out, *pos. 2* shoulder in, *pos. 3* midline, *pos. 4* x-midline—where the shoulder refers to the shoulder of the corresponding hand’s arm (please see in text for more details)

Our previous work (Dempsey-Jones and Kritikos 2017) suggests that drift in the RHI varies as a function of hand usage, with maximal drift in the location of space where the arm operates most frequently—near the shoulder of origin (Howard et al. 2009). If this spatial modulation of drift truly results from preferential use and increased habitual action, we may see a stronger modulation of drift for the dominant hand with respect to the action space. Specifically, we predicted that—for both the left and right hand, of left- and right-handers—we would see greatest drift near the shoulder, reducing as the hand moved laterally towards, and across the midline. We anticipated there would be sufficiently extreme variation in drift across space to cause a significant linear effect of drift for the dominant hand (see Prediction, Fig. 1a). In contrast, we expected a less significant variation in drift across space in the non-dominant hand—resulting in a reduced, or even non-significant linear effect of drift (see Prediction, Fig. 1b). We aimed to provide support for the role of action in modulating multisensory integration in two ways: first, by revealing patterns of action within a hand shape drift, and second, that activity differences between hands also shapes drift. Exploring such questions provide important steps towards developing our understanding of how human sensorimotor experience guides the formation of a coherent sense of self from disparate sensory inputs.

## Methods

### Participants

Sixty-six students from the University of Queensland were recruited and participated for course credit. All had normal or corrected to normal vision. Ethical approval for the study was provided by the Behavioural and Social Sciences Ethical Review Committee of the University of Queensland (approval code: 11-PSYCH-PHD-06-JS). Thirty-five participants were in the right hand used group (17 males, *M*_age_ = 18.5 years, SEM = 0.26). Thirty-four were in the left hand used group, though due to recording error four were removed, leaving 30 (12 males, *M*_age_ = 19.2, SEM = 0.49). Participants were recruited via separate advertisements targeting people who self-reported as either left- or right-handed (depending on the group currently being recruited). Upon presentation for the experiment, the degree of handedness was recorded using a questionnaire (see below). Some analysis on this sample has previously been published, though on questions unrelated to handedness (see Dempsey-Jones and Kritikos 2017 for more details).

### Edinburgh Handedness Inventory (EHI)

Handedness was assessed by way of the EHI (Oldfield 1971), which gathers information regarding the hand participants use to do a series of daily tasks. Based on this information, participants were divided into binary handedness groups: they were coded as left-handed if they scored between − 100 and 0, and right-handed if they scored between 0 and 100. For the 30 left-handers, 16 were randomly assigned to have the illusion performed on their left hand and 14 on the right hand. For right-handers, 19 had the illusion on the right and 16 on the left hand (both handedness variables were, thus, varied between groups).

### Experimental apparatus

**Fig. 2:**
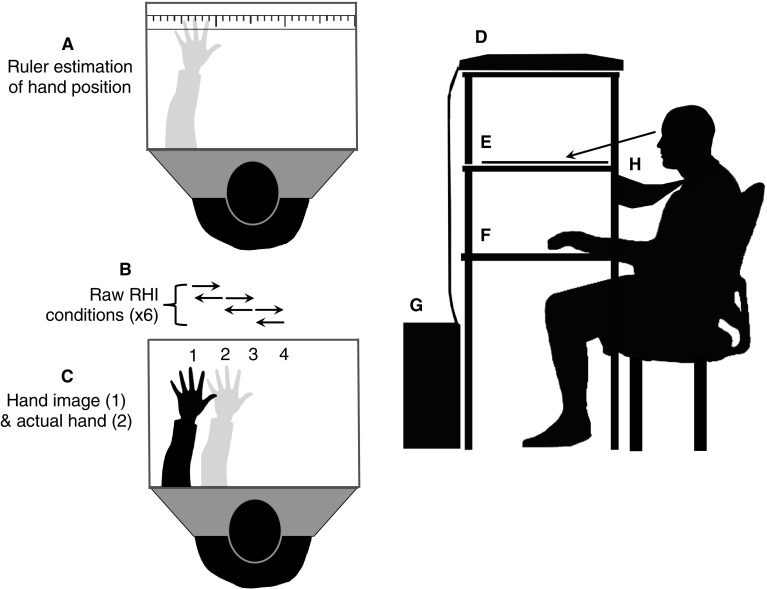
Apparatus and experimental conditions. **a** Ruler used for estimation of hand position (note: hand was not visible, but is presented here to demonstrate how finger position estimations were made). **b** Arrows demarcating the combination of the six raw RHI conditions into the four hand position conditions. The RHI induction in any one condition would shift felt hand position from the location of the arrow end, to the position of the arrow tip—as in the example **c** showing shift from the actual hand location (position 2) to the hand image position (1), again the actual hand position was not visible. **d** Computer screen used to present stimuli. **e** Mirror used to reflect stimuli on the screen above—making hand image appear as if it was located below the mirror in the same spatial depth plane as the actual (hidden) hands. **f** The experimental workspace where the participant’s actual hand was placed. **g** Computer tower. **h** Cover used to prevent vision of the participant’s body

We used a specialised apparatus that allowed realistic hand images to be presented in the spatial depth plane of the actual hand, as opposed to a traditional rubber prosthetic hand (Dempsey-Jones and Kritikos 2014; Dempsey-Jones 2016; see Supplementary Materials for more information). This apparatus consisted of three equidistant horizontal shelves (see Fig. 2). The top shelf, which was at head height, had an LCD computer screen fitted into it facing downwards for presentation of stimuli (size, 51 × 33 cm; resolution, 1680 × 1050 pixels).

The image of a single hand (left or right, depending on condition assignment) was presented on screen. This image was reflected by a mirror set into the middle shelf, at chest height. This created the illusion that, when participants looked down into the mirror, they were looking down at their own hand through a pane of glass. We ensured participant’s arms could rest comfortably in a pronated position on the bottom shelf (the experimental workspace) by adjusting the chair height. Participant’s upper arms were by their side and their forearms projected at 90° from the body, parallel with the ground (also see Fig. 2). Participants placed their chin on a chinrest to maintain head position. The participant’s body was occluded from them by the apparatus and a cover extending from the apparatus to the body (secured behind the neck) (see Fig. 2h). All stimuli presentation was controlled with Eprime (version 2.0, https://www.pstnet.com/).

### Real hand/ hand image positions

To look at differences in drift over different spatial locations we placed the participant’s hand in one of four positions. These positions were located 10 cm apart, on a straight lateral plane across the bottom shelf of the apparatus (perpendicular to the mid-sagittal plane; see Fig. 2c). The positions shoulder out and shoulder in were located 5 cm either side of the shoulder of the hand being used for the illusion (outside and inside, respectively). Visual angles of these positions were 25.73° and 14.56° from straight ahead, in the direction of the shoulder of origin, respectively. The Mid (‘midline’) position was at the body midline (0°). The x-mid (‘cross-midline’) position, was the mirror image of shoulder in, and was located 14.56° from straight ahead, in the direction of the other shoulder (of the arm not being subjected to the illusion).

We conducted pilot work to ensure the hand positions were as close as possible to the desired bodily locations for the average participant, and were naturalistic and comfortable to maintain because extreme joint positions that cause discomfort reduce proprioceptive localisation (Rossetti et al. 1994). However, given natural population variation, the hand position–body part correspondences were approximates for individual participants. Future work may avoid noise introduced into the data by such variations by calibrating hand positions for individual participants, ensuring the best matching possible with the shoulder(s) and midline, etc., though unfortunately time and equipment constraints did not permit this to be done here.

We carried out the RHI between all pairs of adjacent positions. This created six ‘raw’ illusion conditions, each of which were repeated twice, creating 12 trials total (order randomized; see Fig. 2b for details on how the raw conditions were combined into the four critical hand position conditions used for analysis). We were limited to two repetitions due to the large number of conditions in the current study. Despite a small number of trials per condition, however, our data had good reliability (please see Supplementary Methods for statistics).

### Estimation of proprioceptive hand position

To estimate drift, we measured changes in the perceived position of the middle finger of the hand used for the illusion. Participants estimated the position of their (hidden) middle finger using one of 15 different rulers with veridical millimetre demarcations displayed on the computer screen, presented at the same height and depth as the real hidden hand (see Fig. 2a). Participants verbally reported the number best corresponding with their middle finger position, which was coded into the computer by the experimenter. Please note, here we did not measure subjective outcomes of the RHI, e.g., embodiment changes (Ehrsson et al. 2005; Longo et al. 2008; Tsakiris et al. 2007) as our interest was in proprioceptive changes alone, known to critically depend on processes of multisensory integration (see “Discussion” regarding the dissociation between subjective and drift RHI outcomes).

### Modified RHI induction

The RHI induction traditionally involves synchronous stroking of the participant’s own hand and the rubber hand (Botvinick and Cohen 1998; Longo et al. 2008; Tsakiris and Haggard 2005). This ‘intermodal matching’ has been suggested to be causal in illusion induction because it creates a three-way interaction between the visual, touch and proprioceptive information, which causes subjective RHI outcomes, that in turn cause proprioceptive drift (Botvinick and Cohen 1998). In apparent support of this, many studies report a reduction or attenuation of illusion under asynchronous stroking, as compared to synchronous stroking (Botvinick and Cohen 1998; Longo et al. 2008; Tsakiris and Haggard 2005; Zopf et al. 2010).

However, the causality of synchronous stroking in causing the RHI has been questioned following results demonstrating greater proprioceptive drift in a condition with no stroking (as compared to both synchronous, and asynchronous stroking; Rohde et al. 2011), and other studies showing drift without tactile matching (Durgin et al. 2007; Holmes et al. 2006). Thus, intermodal matching may not cause drift, but mismatched inputs may disrupt it (Rohde et al. 2011). In the current study, we were interested in whether handedness modulates drift, rather than whether it could be attenuated with mismatched stroking. Given this, and the unresolved influence of asynchrony in disrupting drift, we chose not to include asynchronous conditions in our design.

While the intermodal matching may not produce drift, it should not reduce/attenuate it. Thus, to be in line with other comparable research, we included synchronous stimulation during illusion induction (see also Dempsey-Jones and Kritikos 2014, 2017). This was achieved by way of stroking the real hand and hand image concurrently with a paintbrush at approximately 1 Hz. Both brushes were affixed to the apparatus to ensure angle, pressure and contact of the brushes to be stable over time.

### Procedure

At the beginning of each trial, the experimenter placed the participant’s hand in position. A ruler appeared on-screen, and participants made their pre-RHI position estimation (procedure above). The ruler disappeared, and the RHI was induced over a 60 s period (i.e., the spatially displaced hand image position was presented on screen, and synchronous stimulation was applied). The induction stopped, and then participants made their post-illusion position estimation—followed by a 60 s inter-trial interval (ITI). During the ITI, participants placed their hand back onto their lap.

### Analyses

We used a pre-to-post induction difference score as our measure of drift (significant change in position was observed in all six conditions, *p* < 0.001). We first used mixed model ANOVAs to demonstrate that both groups showed a difference in spatial drift effects between hands. We then used linear contrasts to determine the nature of these spatial drift effects for each group and hand separately (Dempsey-Jones and Kritikos 2017). Linear contrasts (which fall within the framework of the ANOVA) were used because they provide a powerful tool to look for a priori effect types, e.g., as here first-order linear effects, or for higher-order effects such as quadratic or cubic functions (Abdi and Williams 2010; Seltman 2013). Particularly, here we use linear contrasts to ask whether drift is maximal near the shoulder of origin, decreasing in a linear manner with distance from this position (rather than using a battery of post hoc *t* tests comparing drift at each hand position separately, which runs into significant issues of multiple comparisons). All statistical analyses were run on SPSS, version 22 (IBM Corp, Armonk, NY, USA).

Please note, discussion of ‘hand position’ conditions refers to the location of the participant’s actual hand (as opposed to the position of the hand image).

## Results

We first looked at the left-handed participants to describe the particular spatial patterns of drift they show across the left and right hands. Please note, left- and right-handers were analysed separately as the directly opposing linear drift effects the two groups show across hands would cancel out, eliminating spatial drift effects.

**Fig. 3:**
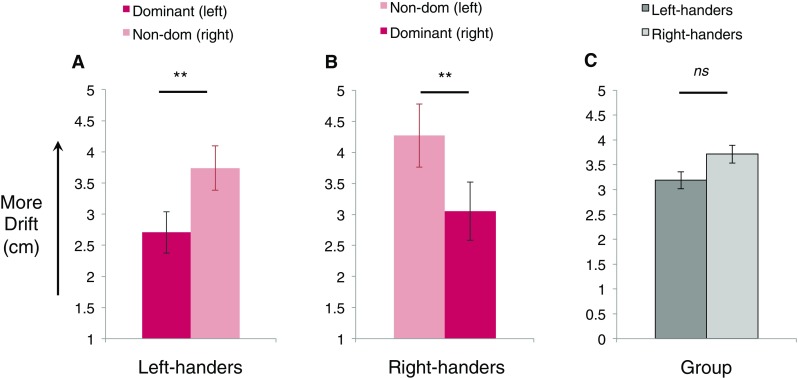
**a, b** Main effects of drift magnitude across handedness groups and hand used. Results revealed there was significantly less proprioceptive drift for the dominant hand (dark) compared to the non-dominant hand (light) for both left- and right-handers. **c** Comparison of overall drift susceptibility in left- (dark) and right-handers (light) was unable to reveal a significant difference when collapsing over hand used

Overall, as predicted we found that left-handers showed greater drift in the non-dominant hand, compared with the non-dominant hand. Also consistent with our predictions, we found a difference in the linear pattern of drift between the two hands. This was revealed by a 4 × 2 mixed ANOVA with within-participants factors hand position (four levels: shoulder (out), shoulder (in), midline, x-midline), and between-participants factor hand used (two levels: left hand used, right hand used). This analysis returned a significant main effect of hand used (*F*(1, 28) = 4.49, *p* = 0.043, *η*_p_^2^ = 0.14). Inspection of the means allowed us to interpret this main effect: collapsing across all four hand positions, there was indeed more drift overall for the non-dominant (right) hand (*M* = 3.74, SEM = 0.36) compared with the dominant (left) hand (*M* = 2.71, SEM = 0.33). See Fig. 3a.

**Fig. 4:**
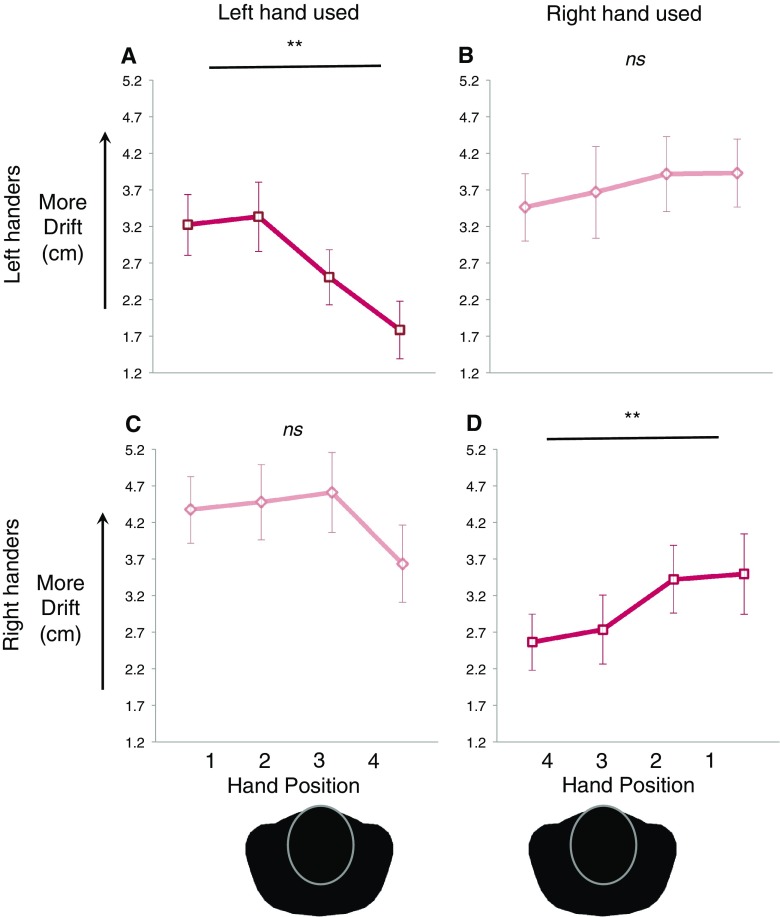
Handedness and the spatial modulation of drift. For both left-handers (top row) and right-handers (bottom row), we found there was generally more drift when an arm was in its habitual action space, i.e., near the left shoulder when the RHI was induced on the left hand, and near the right shoulder for the right hand. For both handedness groups, we found a significant linear effect of drift only for the dominant hand (**a, d**), but not for the non-dominant hands (**b, c**). *Pos. 1* shoulder out, *pos. 2* shoulder in, *pos. 3* midline, *pos. 4* x-midline—where the shoulder refers to the shoulder of the corresponding hand’s arm (please see in text for more details)

The above ANOVA also returned a significant linear interaction of hand position × hand used, *F*(1,28) = 7.34, *p* = 0.011, *η*_p_^2^ = 0.21 (see Table 1(A) for lower-order and non-significant effects). Subsequently, we performed a single repeated-measures ANOVA with within-participants contrasts for each hand separately. This analysis had a single factor of hand position (four levels: shoulder (out), shoulder (in), midline, x-midline). This revealed that, for the dominant (left) hand of left-handers, there was a significant linear effect of hand position (*F*(1,15) = 21.14, *p* = 0.021, *η*_p_^2^ = 0.31; see Fig. 4a). Examining the mean values to determine the direction of this linear effect indicated that the greatest drift occurred when the hand was positioned near to the left shoulder—decreasing as the hand moved laterally to the right.

**Table 1:** Full results for statistical comparisons described in the main text

	Left-handers			Right-handers		
	A	B	C	D	E	D
Comparison	Mixed ANOVA: left vs. right hand used	Linear contrasts: left hand used	Linear contrasts: right hand used	Mixed ANOVA: left vs. right hand used	Linear contrasts: left hand used	Linear contrasts: right hand used
Hand position	*F*(1,28) = 1.92, *p* = 0.177, *η*_p_^2^ = 0.06	*F*(1,15) = 21.14, *p* = 0.021*, *η*_p_^2^ = 0.31	*F*(1,13) = 1.41, *p* = 0.258, *η*_p_^2^ = 0.10	*F*(1,33) = 0.29, *p* = 0.597, *η*_p_^2^ = 0.01	*F*(1,18) = 1.05, *p* = 0.319, *η*_p_^2^ = 0.06	*F*(1,15) = 5.19, *p* = 0.038*, *η*_p_^2^ = 0.26
Hand used	*F*(1, 28) = 4.49, *p* = 0.043*, *η*_p_^2^ = 0.14			*F*(1,33) = 4.96, *p* = 0.033*, *η*_p_^2^ = 0.13		
Hand position × hand used	*F*(1,28) = 7.34, *p* = 0.011*, *η*_p_^2^ = 0.21			*F*(1,33) = 4.50, *p* = 0.041*, *η*_p_^2^ = 0.12		

Mixed ANOVAs comparing the RHI for left and right hand use conditions for (A) left-handers and (D). right-handers. Follow-up linear contrasts analyses then looked for significant linear effects of drift across the four hand positions, again for (B) the left (dominant) hand used and (C) right (non-dominant) hand used conditions for left-handers, then for (E). The left (non-dominant) and (F) right (dominant) hand used conditions for right-handers. For both groups, a significant linear effect of drift was found for the dominant, but not non-dominant hands. *p* < 0.05 are represented with an asterisk (*)

In contrast, while there was a general visual trend for the opposite pattern in the right hand, this did not reach significance. This was indicated by an ANOVA with the same factor as above, with no significant linear effect for hand position (*F*(1,13) = 1.41, *p* = 0.258, *η*_p_^2^ = 0.10; see Fig. 4b). No higher-order function showed a significant fit for either hand (quadratic or cubic, 0.310 < *p* > 0.859).

We then investigated whether these spatial drift relations held in right-handed group. To summarise, consistent with the pattern seen in left-handers, we found more drift overall for the non-dominant hand, and a difference in the spatial pattern of drift between hands. This was indicated by a 4 × 2 mixed ANOVA with factors hand position (four levels: as above), and hand used (two levels: as above). This analysis returned a significant main effect of hand used (*F*(1,33) = 4.96, *p* = 0.033, *η*_p_^2^ = 0.13). Group means indicated the direction of this main effect: there was greater drift for the non-dominant (right) hand (*M* = 4.27, SEM = 0.37) as compared to the dominant (left) hand (*M* = 3.05, SEM = 0.40; see Fig. 3b).

The above ANOVA also returned a significant interaction of hand position × hand used, *F*(1,33) = 4.50, *p* = 0.041, *η*_p_^2^ = 0.12 (see Table 1(A) for lower-order and non-significant effects). Linear contrasts were conducted to follow up this interaction. Consistent with the left-handed group, linear contrasts revealed a significant linear effect of drift when right-handers had the illusion performed on their dominant (right) hand (*F*(1,15) = 5.19, *p* = 0.038, *η*_p_^2^ = 0.26; see Fig. 4d). Also as with the left-handers, the spatial drift effect was not significant when the illusion was performed on the non-dominant (left) hand (*F*(1,18) = 1.05, *p* = 0.319, *η*_p_^2^ = 0.06; see Fig. 4c). Overall, results from both groups and both hands support our prediction of a significant action-based modulation of drift for the hand that is used more frequently in daily action.

### Is drift magnitude greater for left- or right-handers?

Finally, we collapsed drift across all conditions (all four spatial positions and both hands used) to determine whether there was any evidence for an overall difference in drift magnitude between left- and right-handers, which would suggest a difference in illusion susceptibility between handedness groups. Despite a visual trend (see Fig. 3c), a between-participants *t* test that showed there was no significant difference in the amount of drift shown by left- or right-handers, *t*(63) = − 1.33, *p* = 0.185, Cohen’s *d* = 0.34. Bayesian analysis of this difference (conducted using SPSS Statistics for Mac, Version 25.0, IBM Corp, Armonk, NY, USA) returned a Bayes factor of 2.36, indicating an inconclusive outcome: the data neither provided strong support for a null difference between groups, or evidence in support of a difference between groups (note: the default prior was used for comparison, representing a uniform distribution).

## Discussion

In the current study, we used the rubber hand illusion (RHI) to investigate the link between handedness and multisensory integration of body information, as reflected in proprioceptive drift. We found evidence that handedness affects drift in two specific ways. First, there was less drift overall for the dominant than the non-dominant hand, for both left-handers and right-handers. Second, we found the spatial pattern of drift varied as a function of hand dominance. Specifically, when the RHI was performed on the dominant hand, there was a significant linear change in the amount of drift when the hand was in different positions laterally across the body, away from shoulder of origin. This difference was not seen for the non-dominant hand (where the linear difference in drift did not reach significance). Finally, we were unable to provide evidence for a difference in the total amount of drift between left- and right-handed groups (when collapsing across hand used), suggesting there may be no inherent susceptibility to the illusion as a function of handedness—or if there is, this difference is particularly small (as it did not reach significance despite our large sample size, *N* = 65).

### Reduced drift magnitude for the dominant hand

We suggest our demonstration of reduced drift for the dominant hand could be interpreted in line with the principles of optimal integration theory. This theory suggests multisensory information is integrated as a function of the reliability of the sensory inputs (Ernst and Banks 2002; Ernst and Bülthoff 2004). In the RHI, there is a spatial separation between the felt location of the actual hand and seen position of the rubber hand (or hand image, as in the current experiment). Following illusion induction, the felt position of the hand is shifted towards the seen position, i.e., proprioceptive drift occurs (Botvinick and Cohen 1998; Costantini and Haggard 2007; Dempsey-Jones and Kritikos 2014, 2017; Rohde et al. 2011; Tsakiris and Haggard 2005), with more drift indicating stronger integration of inputs (Botvinick and Cohen 1998). This change occurs because visual information is more reliable than proprioceptive information, due to its heightened spatial acuity (van Beers et al. 1998, 1999).

Increased frequency of dominant hand usage in the performance of daily activities, particularly those requiring fine discrimination (Bryden et al. 2000; Oldfield 1971) may lead to greater reliability of proprioceptive information for the dominant hand through incidental ‘training’ (Barnsley and Rabinovitch 1970; Ni Choisdealbha et al. 2011). Increased reliability of proprioceptive information for the dominant hand could lead to a greater weighting for proprioception in integration, and subsequently, reduced integration of the false visual information with the felt information—revealed by less drift (Rohde et al. 2011). These results fit with those from the tendon vibration illusion (perceived movement of a stationary limb due to vibratory stimulation; Sittig et al. 1985; Tidoni et al. 2015), which is of greater magnitude and faster onset for the non-dominant arm (Tidoni et al. 2015). Together, these results suggest that there may be less representational plasticity in response to somatosensory illusions in the dominant than the non-dominant hand, which may indicate resistance to changes in circumstance required for behavioural stability.

### Handedness and the spatial modulation of drift magnitude

We previously showed that drift is maximal in the habitual action space of the arm, that is, when the hand is near the shoulder of origin (Dempsey-Jones and Kritikos 2017). Here, however, we show a significant modulation of drift with proximity to the action space for the dominant hand alone. In our previous study, we did not identify an effect of handedness on the spatial drift effect because, critically, we did not examine left-handed and right-handed groups separately for the left and right hands. Thus, we were unable to identify that the spatial effect varied between dominant and non-dominant hands. The current study, therefore, extends this previous work to reveal further details about how action may influence multisensory integration in ways that are more specific and nuanced than previously conceptualised. Interestingly, a recent study by Smit et al. (2017) did not find any relationship between handedness and the hand used for RHI on drift magnitude. We suggest the divergence in results may stem from the immersive RHI induction apparatus and real hand images used in the current study providing a stronger illusion, allowing more power to detect this drift effect.

But why might a concentration of hand action around a particular area lead to greater integration of visual and proprioceptive body information in this space? It is known that the integration of multisensory inputs improves accuracy and speed of perception when compared to single sensory input sources (reviewed in Calvert et al. 2004). This is reflected in the properties of ‘superadditive’ multisensory neurons that show larger responses to multisensory stimulation than the sum of the response to individual sensory components alone (Stein and Meredith 1993). It may be that the requirement for precision where the hand most commonly performs manual actions leads to a greater integration in this area, to improve perception and task performance. In the case of the RHI, greater integration of visual and proprioceptive inputs in this space leads to a stronger illusion—due to the spatial displacement of visual and proprioceptive inputs (see above). In this specific and unusual case, it may be that the mechanism is subverted to cause less accurate perception of the hand location in this space (more drift), where typically it would cause enhanced perception by integration.

As stated above, our results suggest this action-based effect is significant for the hand that is used more frequently in action, the dominant hand. This dominance-related finding is consistent with those from the popular hand laterality paradigm (introduced by Parsons 1987, 1994). In this task, identification of the laterality of a hand stimulus (‘left or right?’) is slowed when the stimulus is presented at more extreme rotations, e.g., 0° or 180° (Brady et al. 2011). While various factors have been shown to modulate this laterality effect, these modulatory effects often only hold for the dominant hand, e.g., pinched vs. open hand positions (Meugnot et al. 2015), and other tasks (Ni Choisdealbha et al. 2011; Parsons 1987). Because performance on the task has been suggested to rely on retrieval of the internal motor model for the seen hand (Brady et al. 2011) as well as motor imagery (Parsons 1987, 1994), this suggests activation of dominant hand representations causes greater engagement of sensorimotor processes. Similar effects have been seen in other tasks. For instance, Hoover et al. (2016) showed superior detection of movement for self vs. other hand stimuli, only in the dominant hand. They attributed this to enhanced ownership and embodiment of the dominant hand, suggesting there may also be a role for subjective aspects of body representation in handedness effects. Therefore, a variety of lower- and higher-level factors might combine to cause stronger effects for the dominant hand across paradigms (also see Gandrey et al. 2013 for improved mentally simulated actions in the dominant hand of right-handers).

### Non action-related interpretations

Given that we show reduced drift for the more frequently used (dominant) hand, it may be expected that we would see more drift in area of space that is more frequently engaged in action (i.e., the habitual action space, near the shoulder of origin). In contrast, here we see the reverse. That is, more action with the dominant hand leads to less drift—and more action near the shoulder leads to more drift. While we posit our two findings converge in that they both suggest drift modulation occurs as a function of habitual hand use, this apparent contradiction suggests the precise mechanism relating action and integration may remain to be determined. While our interest here lies in action-based effects on integration, it is now being increasingly recognised that causal inferences about perceptual signals influence sensory perception and processing (Kording et al. 2007; Shams and Beierholm 2010). In this way, visual plausibility might affect the integration of visual and proprioceptive inputs to shape drift. Specifically, if the hand operates more frequently in a particular area of space, this might alter computation regarding the reliability of visual or felt inputs and influence their integration. This is consistent with previous findings showing that visual rotation of a rubber hand (into an anatomically impossible position), causes reduced drift (Costantini and Haggard 2007). Finally, it may also be important to consider potential differences in action-based effects on multisensory integration demonstrated between hands, as opposed to those occurring across space.

In all, here we demonstrate handedness modulates drift within hands (spatial modulation of drift) as well as between hands (difference between dominant and non-dominant hands). Future work will be required to disambiguate usage-based, visual expectation/ cognitive and potential possible laterality-based (see “Introduction”) contributions to these handedness-based drift effects. Ideally, training protocols of new or extreme spatial action patterns may provide causal evidence of the relationship we propose here.

### Handedness and the RHI: previous research

In addition to drift, the RHI is known to also cause striking shifts in self-reported ownership and embodiment—which are increased for the rubber hand, and decreased for the participant’s own hand after illusion induction (Botvinick and Cohen 1998; Costantini and Haggard 2007; Ehrsson et al. 2005; Tsakiris et al. 2007; Preston 2013). These changes are thought to reflect subjective incorporation of the rubber hand into the body representation as a result of the illusion (Botvinick and Cohen 1998). Somewhat surprisingly, given our results and the relationship between handedness and various biases of body representation (reported in the “Introduction”), these other studies did not report a relationship between subjective RHI outcomes between left- and right-handers (Ocklenburg et al. 2011; though see; Smit et al. 2017 who report an interaction between handedness and subjective illusion that was not followed up due to inconsistency with experimental predictions). However, Niebauer et al. (2002) report less subjective illusion in more strongly handed people compared with mixed handed people. They suggest reduced interhemispheric communication in strongly handed people leads to less left/ right hemisphere updating and, thus, greater susceptibility to these biases. While interesting, these results cannot inform on the question of handedness and drift because, while often considered measures of the same outcome, subjective and drift outcomes of the RHI have been shown to index correlated, but distinct, aspects of self-representation, supported by dissociable neural substrates (Dempsey-Jones and Kritikos 2013; Fiorio et al. 2011; Holle et al. 2011; Honma et al. 2014; Longo et al. 2008; Rohde et al. 2011).

### Limitations

While our experiment tested a large sample of 65, we were limited to around 15–19 people per individual condition (of handedness group, and hand used). This meant that we may have been lacking statistical power to detect a small linear effect of drift that existed in the non-dominant hands of either group. Demonstration of a significant linear effect for the non-dominant hand would be aligned with our general predictions (in that we expect for both hands we should see maximal drift in the habitual action space). As stated in the “Introduction”, we predicted the more used (dominant) hand would show a significant usage effect, where the lesser used (non-dominant) hand would either show a slight or non-significant modulation of drift by use. This may be further explored by future research that, for example, looked only at one handedness group—allowing more participants in each ‘hand used’ condition.

## Conclusions

In sum, here we found that handedness was an important factor affecting the integration of multisensory body information, as reflected in drift patterns. We showed that there was reduced drift for the dominant (as compared to non-dominant hand) hand, which is likely due to greater stability of proprioceptive information causing resistance to illusory shifting. We also found a significant linear effect of drift for the dominant hand only, which appears to be consistent with elevated hand preference in action. Considered in combination, these results support the idea that multisensory integration varies as a function of habitual patterns of bodily action. Previous research has demonstrated greater multisensory integration in the peripersonal space, i.e., within the reach of the arms, as contrasted to extrapersonal space (Brozzoli et al. 2009; Canzoneri et al. 2012; Holmes et al. 2007; Làdavas et al. 1998). In the light of our results, we suggest multisensory integration may be better described as varying with respect to regions of space that are functionally relevant for human behaviour, as opposed to simply varying with proximity or distance from the body.

## Electronic supplementary material

Below is the link to the electronic supplementary material.

Supplementary material 1 (DOCX 17 KB)
