# Supplementary material for: Handedness modulates proprioceptive drift in the rubber hand illusion
Source: Exp Brain Res. 2018 Nov 8;237(2):351–61. doi: 10.1007/s00221-018-5391-3 (PMC6373180; doi:10.1007/s00221-018-5391-3)
Supplement: Supplementary file 1 — Supplementary material 1 (DOCX 17 KB) [file 221_2018_5391_MOESM1_ESM.docx]

**Supplementary Materials**

**Supplementary Methods**

*Data reliability assessment*

Despite a low number of trials per condition (2 repetitions), our data had low noise: this was indicated by a low standard error of the mean (SEM; collapsed across conditions, drift: M = 3.47, SEM = 0.19). To look within-participant variability, we correlated the values for the first and second repetition of all judgements (across condition) made by our participants. The correlation was high (R = .704, SEM = 02), and significantly greater than zero (p < .001) supporting reliability of participants’ judgements. We also showed high between-participants reliability, likely given our large sample size (N = 65). For instance, collapsing across handedness groups, there was no difference between the left and right hand used groups in mean drift (t(63) = 0.46, p = .648), indicating stability in illusion strength between randomly selected groups.

*Additional information: Hand images used for illusion induction*

The hand images were photos taken using a representative pilot participant’s hand placed on the experimental apparatus. One image was taken in each of the four positions, from the vantage point of the middle of the computer screen. This was done because relative rotation of the (real or rubber) hand can create a violation between what is seen and felt, and therefore reduce illusion effectiveness due to anatomical implausibility (Costantini and Haggard 2007). Participants placed their chin on a chin-rest (midway between the middle and top shelf), with their forehead resting against the apparatus to ensure consistency in position and elevation of the head in viewing these images.
